# 中空结构的双金属有机骨架材料作为固相微萃取纤维涂层用于多环芳烃的高灵敏检测

**DOI:** 10.3724/SP.J.1123.2022.05001

**Published:** 2022-11-08

**Authors:** Wenmin ZHANG, Qingqing LI, Min FANG, Lan ZHANG

**Affiliations:** 1.闽江师范高等专科学校, 福建 福州 350108; 1. Minjiang Teachers College, Fuzhou 350108, China; 2.福州大学, 食品安全与生物分析教育部重点实验室, 福建 福州 350116; 2. Ministry of Education Key Laboratory for Analytical Science of Food Safety and Biology, Fuzhou University, Fuzhou 350116, China

**Keywords:** 双金属, 金属有机骨架, 固相微萃取, 气相色谱-串联质谱, 多环芳烃, bimetal, metal-organic frameworks (MOFs), solid-phase microextraction (SPME), gas chromatography-tandem mass spectrometry (GC-MS/MS), polycyclic aromatic hydrocarbons (PAHs)

## Abstract

环境样品中多环芳烃(PAHs)含量较低且样品基质复杂,直接利用仪器进行含量测定比较困难,因此在仪器分析之前需要对环境样品进行必要的前处理。大多数前处理技术的萃取效率取决于萃取材料的特性。目前,金属有机骨架材料(MOFs)作为一种由金属离子与有机配体自组装而成的多孔材料,已经被用作固相微萃取(SPME)的涂层材料应用于PAHs的萃取,但是这些MOFs涂层材料由于目标物较难达到其深层的吸附位点,使得萃取过程往往需要较长的平衡时间;此外,大多数MOFs由单金属离子配位构成,能够提供的开放金属活性位点种类比较单一,较难获得最佳的萃取性能。这些问题在一定程度上限制了MOFs材料在SPME领域的应用。该研究制备了一种中空结构的双金属有机骨架材料(H-BiMOF),并将其作为SPME的涂层材料,用于萃取环境样品中痕量的PAHs。由于中空的结构和双金属的组成,H-BiMOF涂层材料拥有比表面积利用率高、传质距离短等优点,可以使萃取过程快速地达到平衡。同时,双金属的引入提供了种类丰富的金属活性位点,提高了对PAHs这类富电子云目标物的萃取效率。与气相色谱-串联质谱(GC-MS/MS)相结合,建立了一种用于环境水样中PAHs分析的新方法。所建立的分析方法具有检出限低(0.01~0.08 ng/L)、线性范围宽(0.03~500.0 ng/L)、重复性良好(相对标准偏差≤9.8%, *n*=5)等优点,并成功地用于实际湖水样品中7种PAHs的检测。实验结果表明,所建立的分析方法适用于环境样品中PAHs的分析与监测。

## Abstract (en)

Polycyclic aromatic hydrocarbons (PAHs) are among the most harmful persistent organic pollutants that possess high carcinogenicity and teratogenicity; hence, establishing a highly sensitive analytical method for monitoring PAHs in environmental samples is an urgent need. However, due to the low PAHs content in environmental samples and the complex matrix of the samples, it is difficult to directly determine the amount of PAHs using the existing analytical instruments. Therefore, an essential pretreatment of environmental samples should be carried out before instrumental analysis. In most pretreatment techniques, the extraction efficiency depends on the characteristics of the extraction materials. Currently, metal-organic framework materials (MOFs), which are porous materials self-assembled by metal ions and organic ligands, are used as solid-phase microextraction (SPME) coating materials for the extraction of PAHs. However, the following problems limit the application of MOFs in the SPME field: (1) MOF coating materials often require a long equilibration time for extraction because the it is difficult for the target to reach the deep adsorption sites; (2) In addition, most MOFs are formed by the coordination of single metal ions with organic monomers. The single type of open metal active sites is not conducive for realizing high extraction performance. In this study, a hollow bimetal-organic framework (H-BiMOF) was synthesized by the solvothermal method and characterized by transmission electron microscopy (TEM), X-ray diffraction (XRD), nitrogen adsorption-desorption analysis, thermogravimetric analysis, etc. The TEM images and XRD patterns demonstrated the successful synthesis of H-BiMOF with a hollow structure, which was formed through the competitive coordination between benzoic acid and water. The H-BiMOF material showed type-Ⅳ isotherms with a surface area of 1437 m^2^/g and excellent thermal stability. Subsequently, a H-BiMOF-coated SPME fiber was prepared by the physical adhesion method and used to extract trace PAHs from environmental samples. Due to the hollow structure of H-BiMOF, the prepared fibers offer the advantages of high utilization of specific surface area as well as short mass transfer distance, so that the extraction process quickly reaches equilibrium. At the same time, the introduction of bimetals provides a variety of metal active sites, which improves the extraction efficiency of the fiber against electron-rich cloud targets such as PAHs. The prepared fiber also had good service life, with at least 150 cycles. Combined with gas chromatography-tandem mass spectrometry (GC-MS/MS), a new method for the determination of PAHs in environmental water samples was established. Single factor experiments were performed to investigate the effects of the SPME conditions on the analytical performance. Under the optimal conditions, the established method showed low limits of detection (0.01-0.08 ng/L), wide linear range (0.03-500.0 ng/L), good linearity (correlation coefficients≥0.9986), and acceptable reproducibility (relative standard deviations≤9.8%, *n*=5). Finally, typical water samples were analyzed by the established method. Four environmental water samples were collected from Dianchi Lake, Poyang Lake, Taihu Lake, and Xihu Lake in China. No benzo(*a*)anthracene (BaA) and chrysene (CHR) were detected in any of the water samples. However, 17.9 ng/L of fluorene (FLU) and 5.3 ng/L of phenanthrene (PHE) were found in the Poyang Lake sample; 11.3 ng/L of fluoranthene (FLA) and 24.2 ng/L of pyrene (PYR) were found in the Taihu Lake sample; 50.0 ng/L of FLU, 19.5 ng/L of PHE, 14.9 ng/L of anthracene (ANT), 34.2 ng/L of FLA, and 44.5 ng/L of PYR were found in the Xihu Lake sample. The contents of the PAHs detected in all the lake water samples were lower than the Chinese National Standard GB 5749-2006 (2000.0 ng/L). The results of this study indicate that the developed method is suitable for the sensitive detection of trace levels of PAHs in real environmental water samples.

多环芳烃(PAHs)是一种由煤、石油、木材、烟草等有机物不完全燃烧时产生的挥发性烃类化合物,是环境中重要的持久性有机污染物^[1]^。PAHs因高度致癌性和致畸性,已被欧盟和美国环境保护署列入优先控制污染物名单^[2]^。因此,建立一种高灵敏的分析方法用于监测环境样品中PAHs含量十分重要。然而,由于环境样品中PAHs含量较低且样品基质复杂,直接利用仪器进行含量测定比较困难,所以在仪器分析之前需要对环境样品进行必要的前处理。

固相微萃取(SPME)是一种集采样、萃取和浓缩于一体的样品前处理技术,具有操作简单、样品用量小、有机溶剂消耗少、易于与分析仪器联用等优点,在分析领域已经得到了广泛的应用^[3⇓-5]^。SPME过程是基于目标物在纤维涂层和样品溶液之间的分配平衡,因此发展具有高萃取性能的涂层材料对提高萃取效率格外重要。金属有机骨架材料(MOFs)作为一种由金属离子与有机配体自组装而成的多孔材料,具有比表面积大、孔隙率高、组成与结构可调等优点,在分离领域具有广阔的应用前景^[6⇓-8]^。到目前为止,虽然已经有几种MOFs作为SPME的涂层材料用于PAHs的高效萃取^[9⇓⇓-12]^,但是这些MOFs涂层材料由于目标物较难达到其深层的吸附位点,使得萃取过程往往需要较长的平衡时间。此外,大多数MOFs由单金属离子配位构成,能够提供的开放金属活性位点种类比较单一,不能获得最佳的萃取性能。这些问题一定程度上限制了MOFs材料在前处理领域的应用。

综上所述,本实验尝试制备一种中空结构的双金属MOF材料(hollow bimetal-organic framework, H-BiMOF),并将其作为SPME的纤维涂层,用于萃取环境样品中痕量的PAHs。相比于实心结构,中空结构的MOF材料可以拥有更多样化的孔隙结构,更高的比表面积利用率和更短的传质距离,这些特性可以使得萃取过程更快地达到平衡^[13⇓-15]^。同时,双金属的引入可以提供更多种类的金属活性位点,有利于PAHs这类富电子云目标物的高效萃取^[16]^。此外,本实验还评估了H-BiMOF纤维对7种PAHs萃取效果的主要影响因素。最后,将SPME与气相色谱-串联质谱法(GC-MS/MS)相结合,建立了一种用于环境水样中PAHs分析的新方法。

## 1 实验部分

### 1.1 仪器与试剂

TRACE 1300-TSQ 8000 Evo气相色谱-三重四极杆质谱联用仪(美国Thermo Fisher公司); Tecnai G2场发射透射电子显微镜(美国FEI公司); JSM-6300F场发射扫描电子显微镜(TEM, 日本JEOL公司); ASAP 2020氮气吸-脱附测定仪(美国Micromeritics公司); DY5261/Xpert3 X射线衍射分析仪(XRD, 美国CEM公司); DIL 402C热重分析仪(德国Netzsch公司)。

PAHs混合标准溶液购于中国百灵威科技有限公司,该混合标准溶液含有7种PAHs,分别为芴(fluorene, FLU)、菲(phenanthrene, PHE)、蒽(anthracene, ANT)、荧蒽(fluoranthene, FLA)、芘(pyrene, PYR)、苯并[*a*]蒽(benzo(*a*)anthracene, BaA)、䓛(chrysene, CHR)。氯化锆(ZrCl_4_,纯度99.9%)、苯甲酸(纯度99.5%)、内消旋-四(4-羧基苯基)卟吩(TCPP,纯度97.0%)购自中国阿拉丁试剂有限公司。无水乙醇(纯度99.7%)、*N*,*N*-二甲基甲酰胺(DMF,纯度99.5%)、丙酮(纯度99.7%)、氢氟酸(HF,纯度40.0%)、氯化钠(NaCl,纯度99.5%)、氯化锌(ZnCl_2_,纯度98.0%)购自中国国药试剂有限公司。聚酰亚胺密封树脂购自美国Sigma Aldrich公司。实验用超纯水(18.2 MΩ·cm)均由Milli-Q净水器所制备。

### 1.2 标准溶液的配制

PAHs混合标准溶液在4 ℃下避光保存。系列混合标准溶液使用丙酮逐级稀释配制,现配现用。

### 1.3 H-BiMOF材料的合成

H-BiMOF材料通过溶剂热法制备而成,具体过程如下:分别称量1.8 mg的ZrCl_4_、7.0 mg的ZnCl_2_和250.0 mg的苯甲酸加入到2.0 mL的DMF/H_2_O混合溶剂(10:1, v/v)中,磁力搅拌至完全溶解。然后将10 mg的TCCP加入至上述溶液中,继续磁力搅拌10 min。将所获得的均相溶液转移至12 mL的高压反应釜中,在120 ℃下反应24 h。待反应釜自然冷却至室温后,将所获得的产物用无水乙醇洗涤6次(10000 r/min, 5 min)。随后,将产物先后分散在甲醇和丙酮溶液中室温静置24 h,以除去未反应的配体、无机类杂质和DMF等。最后,将产物在120 ℃真空干燥24 h,以获得H-BiMOF材料。

在H-BiMOF材料的优化实验中,保持其他制备条件不变,通过改变Zn^2+^占总金属离子的质量分数(0%、10%、20%、30%、40%和50%),以得到不同Zn^2+^占比的H-BiMOF材料,分别表示为H-BiMOF-*n*(*n*=0、10、20、30、40和50)。

### 1.4 SPME纤维的制备

H-BiMOF材料涂覆的SPME纤维采用物理黏附法制备得到,具体步骤如下:首先,将不锈钢纤维的一端(4.0 cm)浸入到HF溶液中,在70 ℃下刻蚀10 min后,用超纯水和无水乙醇交替清洗干净,以获得一定直径的粗糙表面。随后,在刻蚀过的不锈钢纤维表面涂上聚酰亚胺密封树脂,并迅速在盛有H-BiMOF材料的称量纸上来回滚动,使H-BiMOF材料均匀涂覆在不锈钢纤维表面。最后,将所制得的H-BiMOF涂覆的SPME纤维在250 ℃下老化2 h,以去除材料中吸附的小分子杂质。

### 1.5 样品的制备

4个湖泊水样品分别采集于云南滇池、江西鄱阳湖、江苏太湖和浙江西湖。湖泊水样用0.45 μm滤膜过滤后,储存在棕色玻璃瓶中,于4 ℃的冰箱中保存待用。

### 1.6 顶空固相微萃取(HS-SPME)过程

本实验采用HS-SPME的方式萃取PAHs。首先,将20.0 mL的混合标准溶液或样品加入到25 mL的棕色玻璃顶空瓶中,并使用氯化钠调节溶液盐浓度至15%。然后,将所制备的H-BiMOF纤维插入顶空瓶中,并将其置于数显磁力搅拌恒温水浴锅中进行萃取。待萃取达到平衡后,将纤维从顶空瓶中取出并插入GC进样口,进行GC-MS/MS分析。在SPME过程的优化实验中,所使用的混合标准溶液中FLU、PHE、ANT、BaA和CHR的质量浓度为500 ng/L, FLA和PYR为200 ng/L。

### 1.7 GC-MS/MS分析条件

#### 1.7.1 气相色谱条件

色谱柱:TG-5 MS型石英毛细管柱(30 m×0.25 mm×0.25 μm);载气:高纯氮气(纯度99.999%);流速:1.5 mL/min;进样方式:无分流进样;进样口温度:280 ℃;升温程序:初始温度为50 ℃,保持2.0 min后,以15 ℃/min的速率升温至190 ℃并保持1.0 min,随后以10 ℃/min的速率升温至260 ℃后,立即以5 ℃/min的速率升温至285 ℃并保持5.0 min,总运行时长为29.3 min。

#### 1.7.2 质谱条件

离子源:EI源;四极杆温度:150 ℃;接口温度:280 ℃;离子源温度:230 ℃;碰撞气:高纯氦气(纯度99.999%);碰撞气压:1.5 mTorr;溶剂延迟时间:7.0 min;数据采集模式:选择反应监测(SRM)模式。其他气相色谱和质谱条件(保留时间、定量离子对、碰撞能)见表1。

**表 1:** 7种PAHs的保留时间、定量离子对和碰撞能

Compound	Retention time/min	MS/MS transitions	Collision energy/eV
Fluorene (FLU)	11.81	166.1/165.1	30
Phenanthrene (PHE)	13.77	178.1/151.1	32
Anthracene (ANT)	13.90	178.1/151.1	32
Fluoranthene (FLA)	16.52	202.1/200.1	16
Pyrene (PYR)	17.05	202.1/200.1	16
Benzo(a)anthracene (BaA)	19.96	228.1/226.1	34
Chrysene (CHR)	20.06	228.1/226.1	36

## 2 结果与讨论

### 2.1 萃取材料的优化

H-BiMOF材料是由两种金属离子(Zn^2+^和Zr^2+^)与配体自组装而形成的,而不同的Zn^2+^/Zr^2+^比例会直接影响该萃取材料对PAHs的萃取性能,因此通过预实验探究了最佳的Zn^2+^占比(Zn^2+^占总金属离子的质量分数)。如图1所示,随着H-BiMOF材料中Zn^2+^占比的提高,纤维对PAHs的萃取效果呈现明显的上升趋势;当Zn^2+^占比达到30%时(H-BiMOF-30),对PAHs的萃取效果达到最佳;但随着Zn^2+^占比的进一步提高,纤维的萃取效果却呈现下降趋势。此实验现象可能是因为:(1)随着Zn^2+^占比的增加,H-BiMOF材料拥有了更丰富的开放金属位点,这些金属位点对吸附具有丰富电子云的PAHs起到重要作用^[11]^; (2)在萃取过程中,两种金属位点的吸附作用是相互协调,当Zn^2+^占比大于30%时,过多的Zn^2+^金属位点可能在一定程度上影响了Zr^2+^金属位点的作用,从而降低了材料对PAHs的吸附效果。因此,将Zn^2+^占比为30%的H-BiMOF材料用于随后的实验中。

**图1:**
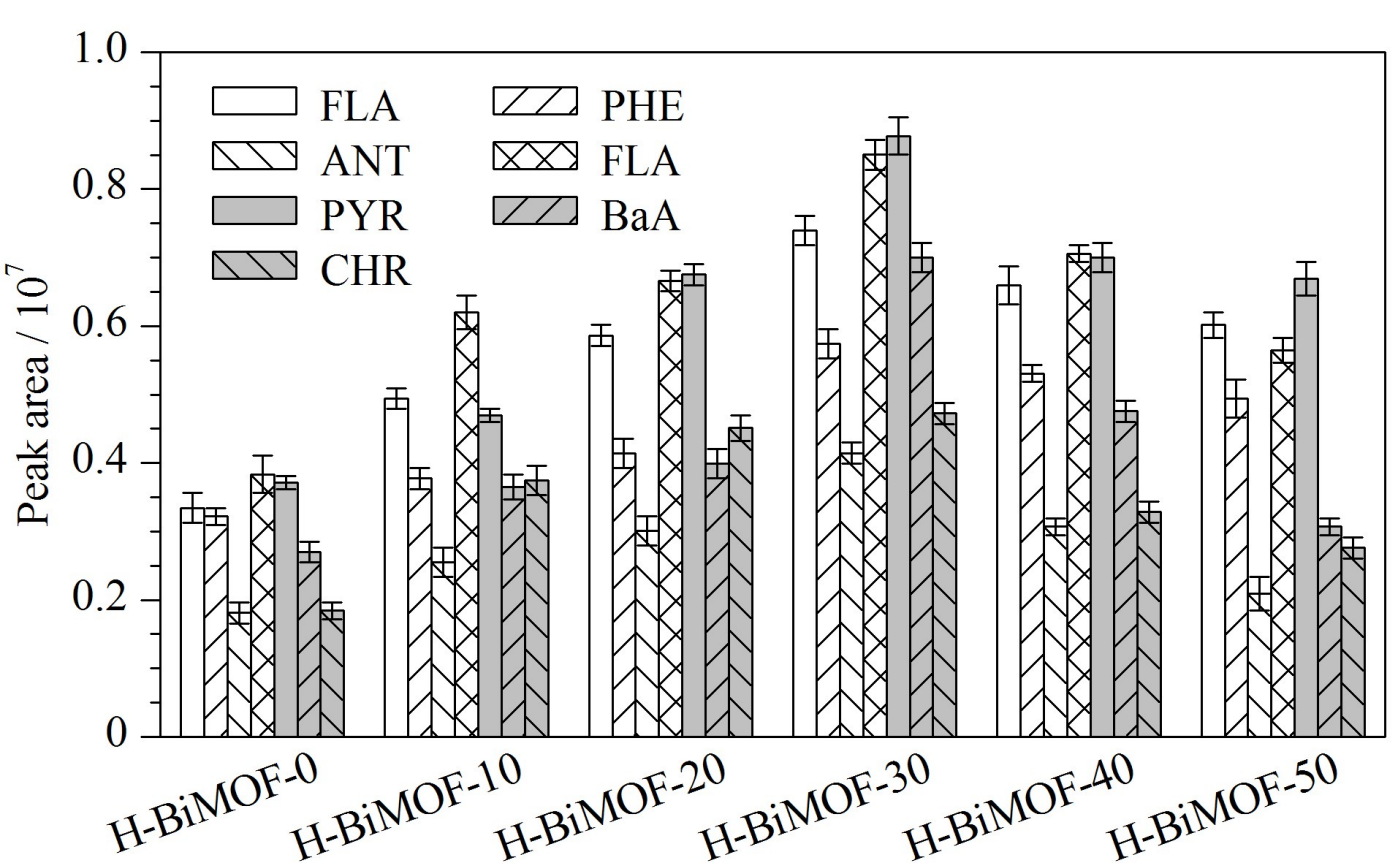
H-BiMOF材料中不同Zn^2+^占比 (0%~50%)对7种PAHs的萃取性能(n=3)

### 2.2 H-BiMOF材料的表征

本实验使用透射电子显微镜和X射线衍射分析仪对所制备的H-BiMOF材料的形貌和晶体结构进行了表征。如图2所示,所制备的H-BiMOF颗粒尺寸均一(约0.89 μm×0.68 μm),晶形良好,并具有独特的中空结构。材料的中空结构是由于苯甲酸与水之间的配位竞争而形成的^[17]^。形成的中空结构可以提供较短的传质距离,有利于萃取更快地达到平衡^[15]^。实验结果表明了H-BiMOF材料的成功制备。

**图2:**
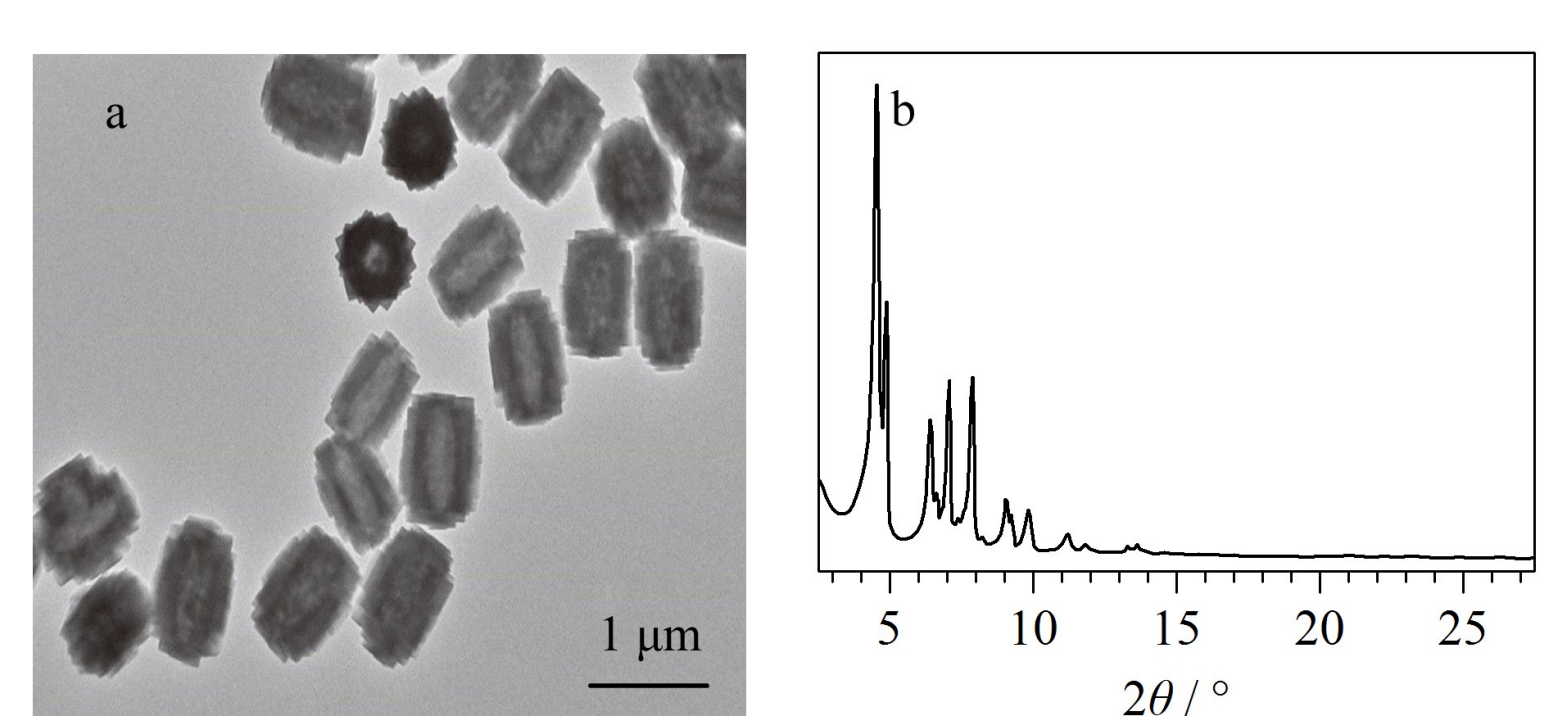
H-BiMOF材料的(a)TEM和(b)XRD的表征图

同时,通过氮气吸附-脱附实验对H-BiMOF材料的多孔结构进行了表征。如图3a和3b所示,H-BiMOF材料呈现出Ⅳ型氮气吸附-脱附曲线,具有较高的比表面积(1437 m^2^/g)以及微孔(1.9 nm)和介孔(4.2 nm)结构,是一种良好的萃取材料。此外,实验对H-BiMOF材料的热稳定性也进行了表征。热重分析(TGA)结果显示,当温度达到300 ℃时,H-BiMOF材料的质量损失不到5%(见图3c),说明其具有良好的耐高温性能,可以满足GC进样口的高温解吸需求。

**图3:**
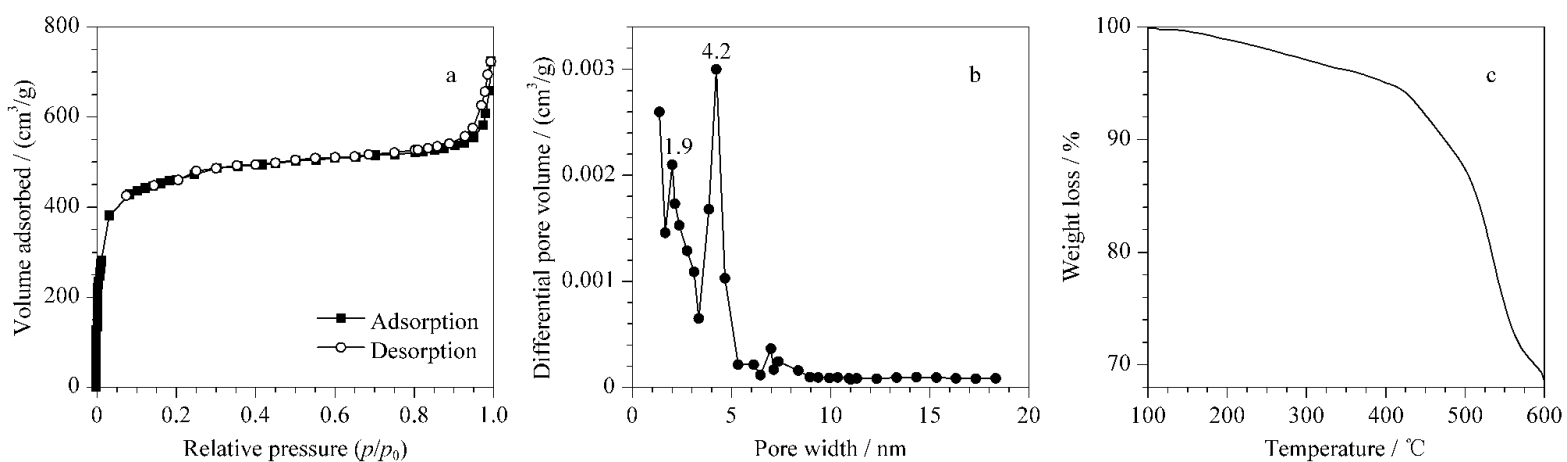
H-BiMOF材料的(a)氮气吸附-脱附等温曲线、(b)孔径分布以及(c)热稳定性的表征图

最后,利用扫描电子显微镜(SEM)对H-BiMOF材料涂覆的SPME纤维进行了表征。如图4所示,在不锈钢纤维表面上涂覆着形貌均一的H-BiMOF材料,并且该材料是由许多纳米立方体堆叠而成的,实验结果表明我们成功制备了H-BiMOF材料涂覆的SPME纤维。

**图4:**
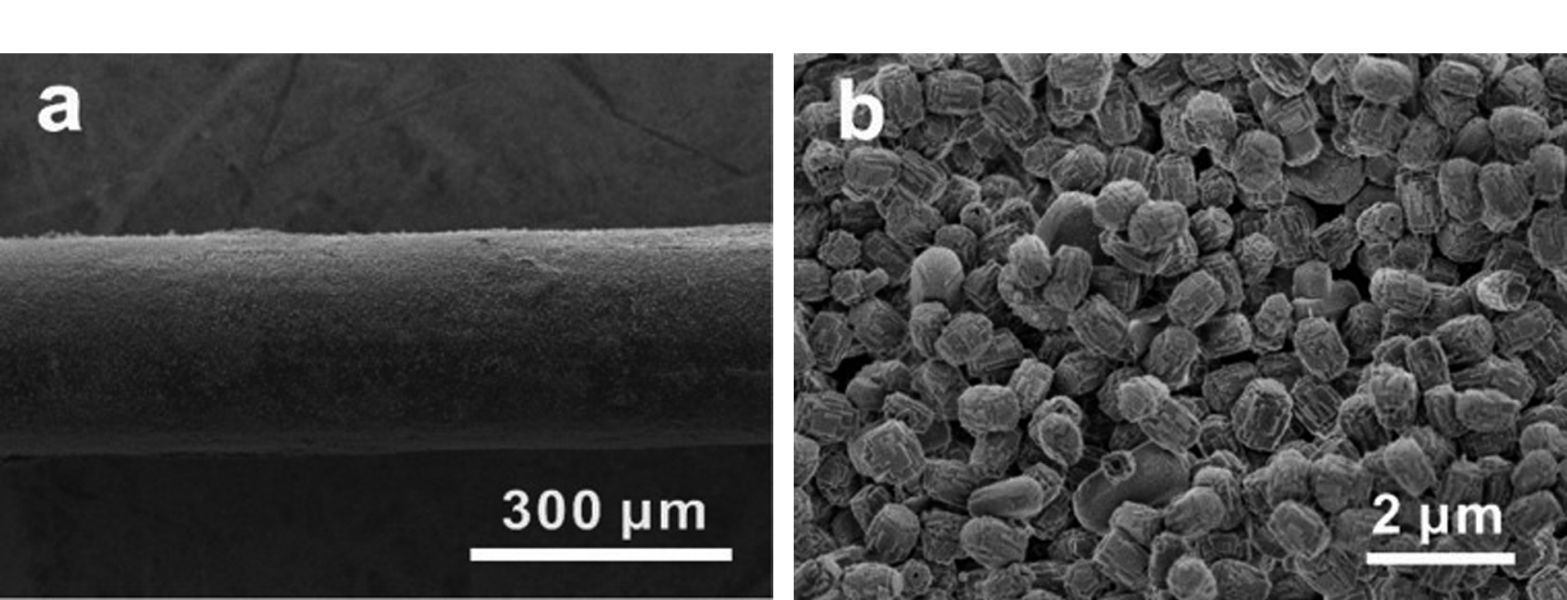
H-BiMOF纤维的(a)低倍镜下和(b)高倍镜下SEM表征图

### 2.3 HS-SPME条件的优化

为了获得最佳的分析性能,通过单因素实验对HS-SPME过程进行了优化,所考察的条件包括萃取温度、萃取时间、盐浓度、解吸温度和解吸时间。

#### 2.3.1 萃取过程的优化

首先,本实验考察了萃取温度在50~70 ℃范围内对萃取效果的影响。如图5a所示,随着萃取温度的升高,萃取纤维对PAHs的萃取效果逐渐提升,并且在60 ℃时达到最大值;但随着萃取温度进一步升高,萃取效果呈现缓慢下降的趋势。这可能是由于萃取温度的升高加快了目标物分子的运动速率,从而提高了萃取效果。然后,吸附又是一个放热的过程,过高的萃取温度反而会降低吸附效果。因此,最佳的萃取温度为60 ℃。

**图5:**
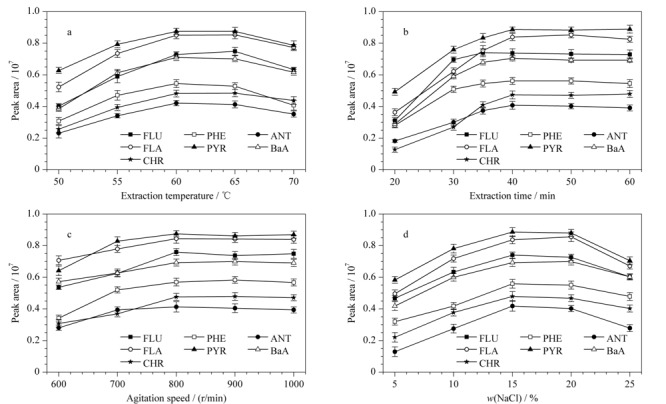
(a)萃取温度、(b)萃取时间、(c)搅拌速度和(d)盐浓度对7种PAHs萃取效果的影响(*n*=3)

随后,实验进一步考察了萃取时间在20~60 min范围内对萃取效果的影响。如图5b所示,随着萃取时间的延长,萃取效果也随之提高,并且在40 min时达到萃取平衡。因此,基于分析效率和方法灵敏度的综合考量,选择萃取时间为40 min以进行后续的实验。同时,实验对搅拌速度也进行了考察。如图5c所示,随着搅拌速度的加快,萃取效果呈现上升的趋势,并在800 r/min时达到最大值。当进一步加快搅拌速度时,萃取效果没有明显的变化。因此,最佳的搅拌速度为800 r/min。

此外,本实验还考察了盐浓度(NaCl的质量分数)对萃取效果的影响。如图5d所示,当盐浓度从5%增加至15%时,萃取效果迅速提高。当盐浓度从15%变化到20%时,萃取效果无明显变化。当盐浓度超过25%时,萃取效果呈下降趋势。这可能是由于盐浓度的提高不仅可以增加溶液在顶空中的蒸汽压,还可以加快目标物向顶空分配。但是,当盐浓度过高时,萃取溶液的黏度和密度过大,不利于目标物的萃取。因此,在随后的实验中将萃取溶液的盐浓度调节为15%。

#### 2.3.2 解吸过程的优化

在解吸过程中,解吸温度和解吸时间是影响萃取效果的重要因素。为了获得最佳的萃取效果,实验先考察了解吸温度在260~300 ℃范围内对萃取效果的影响。如图6a所示,随着解吸温度的升高,萃取效果逐渐提高,当解吸温度为280 ℃时,萃取效果达到最佳。考虑到过高的解吸温度可能会使部分目标物分解,因此,在随后的实验中将解吸温度设置为280 ℃。此外,实验进一步对解吸时间进行了考察。如图6b所示,随着解吸时间从1 min延长至5 min,纤维的萃取效果呈上升趋势。当继续延长解吸时间时,纤维对7种PAHs的萃取效果没有明显变化。因此,最佳的解吸时间为5 min。

**图6:**
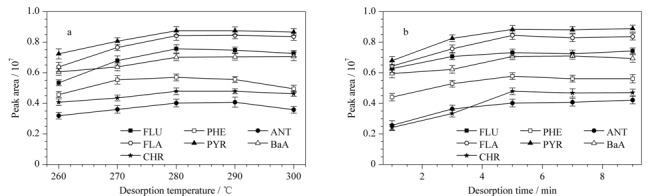
(a)解吸温度和(b)解吸时间对7种PAHs萃取效果的影响(*n*=3)

经过对上述影响因素的考察,获得了最佳的萃取条件:萃取温度60 ℃,萃取时间40 min,搅拌速率800 r/min,盐浓度15%,解吸温度280 ℃,解吸时间5 min。

### 2.4 纤维循环使用性能的表征

循环使用性能是考察萃取纤维是否具有应用前景的重要指标之一。本实验对所制备的H-BiMOF材料涂覆的纤维进行了循环使用次数的考察。如图7所示,在最佳HS-SPME条件下,单根纤维经过150次的循环使用,还能保持良好的萃取效果,对7种PAHs的回收率均在90.0%以上。当使用次数达到250次时,其回收率下降至50%以下。实验结果表明,所制备的萃取纤维具有良好的循环使用寿命,至少可以稳定使用150次。

**图7:**
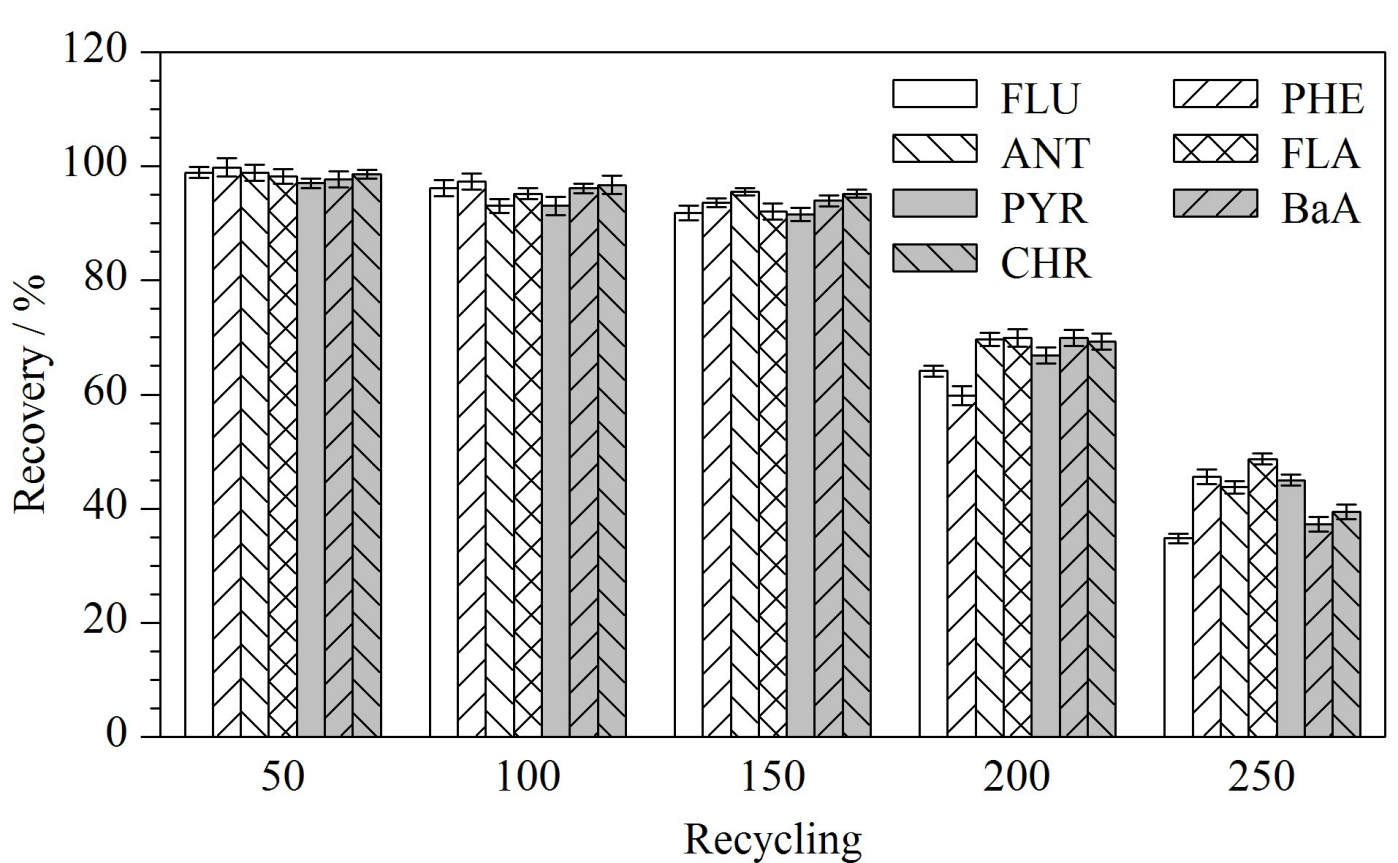
H-BiMOF纤维的循环使用性能(*n*=3)

### 2.5 检测体系的建立与验证

在最佳的萃取条件下,对所建立的HS-SPME-GC-MS/MS方法的性能进行了评估。如表2所示,所建立的分析方法拥有检出限低(0.01~0.05 ng/L)、线性范围宽(0.03~500.0 ng/L)、相关系数良好(*R*≥0.9986)等优点,可以满足实际分析需求。此外,实验对单根纤维的日内和日间精密度进行了评估,RSD分别为2.3%~6.1%和5.6%~7.7%。同时也对不同批次的5根纤维进行了考察,RSD为6.5%~9.1%。实验结果表明所建立的HS-SPME-GC-MS/MS方法具有良好的重复性。

**表 2:** 所建立分析方法的线性范围、相关系数、检出限以及精密度

Compound	Linear range/ (ng/L)	R	LOD/ (ng/L)	RSDs/% (n=5)		
				Single fiber		Fibers of batch-to- batch
				Intra- day	Inter- day	
FLU	0.08-500.0	0.9997	0.03	5.4	7.7	8.1
PHE	0.05-500.0	0.9986	0.02	6.1	6.8	8.6
ANT	0.08-500.0	0.9994	0.03	3.1	5.6	7.8
FLA	0.03-500.0	0.9993	0.01	3.5	7.1	9.1
PYR	0.03-500.0	0.9988	0.01	4.3	6.6	8.3
BaA	0.20-500.0	0.9987	0.08	3.7	6.3	6.5
CHR	0.20-500.0	0.9990	0.08	2.3	5.9	7.4

### 2.6 实际样品的分析

将所建立的HS-SPME-GC-MS/MS方法用于4个湖泊水样中7种PAHs的分析。结果如表3所示,在鄱阳湖水中检测到了17.9 ng/L的FLU和5.3 ng/L的PHE;在太湖水中检测到了11.3 ng/L的FLA和24.2 ng/L的PYR;在西湖水中检测到了50.0 ng/L的FLU、19.5 ng/L的PHE、14.9 ng/L的ANT、43.2 ng/L的FLA和44.5 ng/L的PYR。所有的湖泊水样中检出的PAHs含量均低于国家标准GB 5749-2006(2000.0 ng/L)。为了进一步验证所建立方法在实际样品测定中的可靠性,分别在实际样品中加入低(1.0 ng/L)、中(50.0 ng/L)、高(300.0 ng/L) 3个水平的标准溶液对方法的准确度和精密度进行考察。所得到的加标回收率在81.0%~118.9%之间,RSD小于9.8%(*n*=5)。图8为4个湖泊水样以及其加标水平为100 ng/L的色谱图。实验结果表明,所建立的HS-SPME-GC-MS/MS方法具有良好的准确度和精密度,适用于实际环境水样中痕量PAHs的分析。

**表3:** 7种PAHs在实际湖泊水样中3个水平下的加标回收率及精密度(*n*=3)

Sample	Compound	Background/ (ng/L) (RSD/%)	Recoveries (RSDs) at three spiked levels/%		
			1.0 ng/L	50.0 ng/L	300.0 ng/L
Dianchi	FLU	n. d.	82.3(6.1)	89.3(4.1)	103.5(3.9)
Lake	PHE	n. d.	93.6(7.1)	103.6(8.1)	97.3(5.4)
	ANT	n. d.	108.2(5.2)	89.9(5.4)	90.4(9.8)
	FLA	n. d.	103.2(2.2)	101.8(7.2)	96.7(4.4)
	PYR	n. d.	90.2(5.1)	110.2(5.7)	107.9(6.8)
	BaA	n. d.	94.2(4.8)	94.3(3.8)	99.3(7.8)
	CHR	n. d.	92.2(4.0)	97.2(5.9)	108.8(8.5)
Poyang	FLU	17.9(4.3)	102.9(3.0)	108.9(5.0)	104.9(6.2)
Lake	PHE	5.3(6.6)	107.8(3.9)	117.4(7.9)	106.1(4.5)
	ANT	n. d.	108.1(5.5)	113.1(4.5)	108.3(3.8)
	FLA	n. d.	99.5(4.5)	98.8(4.9)	107.3(5.9)
	PYR	n. d.	100.2(4.4)	107.1(7.4)	97.3(5.9)
	BaA	n. d.	84.3(6.8)	88.3(4.8)	91.0(7.0)
	CHR	n. d.	88.3(3.4)	82.1(6.6)	93.8(8.3)
Taihu	FLU	n. d.	109.5(6.9)	110.3(7.9)	105.3(4.6)
Lake	PHE	n. d.	116.1(4.3)	117.2(9.3)	116.9(8.3)
	ANT	n. d.	84.6(4.8)	86.9(5.8)	94.9(9.1)
	FLA	11.3(3.1)	97.5(7.7)	107.3(7.7)	108.3(5.9)
	PYR	24.2(4.0)	108.9(4.5)	113.9(4.1)	118.9(5.2)
	BaA	n. d.	87.3(6.4)	97.3(4.8)	98.3(3.0)
	CHR	n. d.	107.3(5.6)	108.6(6.8)	107.6(8.8)
Xihu	FLU	50.0(4.3)	103.6(8.9)	108.9(9.3)	106.3(5.3)
Lake	PHE	19.5(7.7)	99.4(7.3)	92.3(6.3)	105.3(4.6)
	ANT	14.9(5.0)	111.3(5.7)	117.3(4.3)	111.1(9.4)
	FLA	34.2(3.2)	108.0(3.8)	118.3(5.8)	114.5(6.3)
	PYR	44.5(2.9)	88.0(6.8)	81.0(4.6)	83.5(3.5)
	BaA	n. d.	96.4(8.9)	90.1(7.0)	93.4(6.8)
	CHR	n. d.	101.7(5.0)	107.6(4.7)	95.4(7.9)

n. d.: not detected.

**图8:**
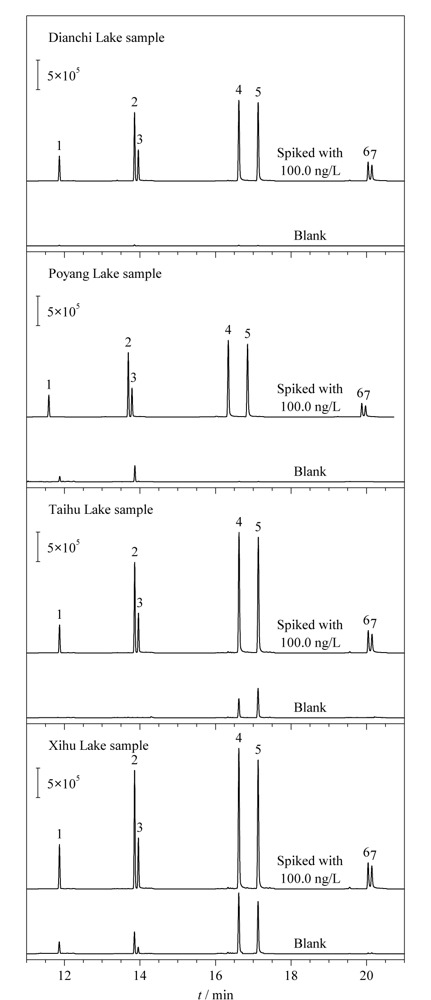
实际湖泊水样和加标湖泊水样的色谱图

### 2.7 与其他方法的对比

将所建立的分析方法与其他文献报道的方法进行比较。如表4所示,本工作所建方法具有线性范围宽、灵敏度高和重复性良好等优点。与其他方法相比较,本方法具有较低的检出限,其得益于H-BiMOF材料对PAHs的高萃取效率。此外,该材料良好的热稳定性和循环使用性能为所建立方法的准确度和精密度提供了保障。这些对比结果表明,本实验所建立的方法是一种简单、高效、灵敏的能用于环境水样中PAHs检测的分析方法。

**表4:** 本方法与其他已报道方法的比较

Coating	Instrument	Linear range/(ng/L)		LODs/(ng/L)		RSDs/%		Ref.
UiO-66	GC-MS	1.0-	5000.0	0.30-	0.60	1.8-	8.9	[9]
Yb-MOF	GC-MS/MS	10.0-	1000.0	0.07-	1.67	2.5-	9.9	[10]
Al-MCM-41	GC-FID	300.0-	6.0×10^5^	60.0-	180.0	1.18-	18.3	[11]
Ni-Zn MOF/g-C_3_N_4_	GC-MS	0.3-	5000.0	0.1-	3.0	3.8-	9.1	[12]
H-BiMOF	GC-MS/MS	0.03-	500.0	0.01-	0.05	2.3-	9.1	this work

UiO-66: one kind of zirconium-based metal-organic framework; Yb-MOF: ytterbium-based metal-organic framework; Al-MCM-41: aluminum-doped mesoporous crystalline material-41; Ni-Zn MOF/g-C_3_N_4_: nickel-zinc-based metal-organic framework/graphitic carbon nitride composite.

## 3 结论

在本实验中,制备了一种具有中空结构的双金属(Zn^2+^和Zr^2+^)有机骨架材料,并将其作为SPME纤维的涂层材料。所制备的纤维不仅对7种PAHs表现出了优异的萃取性能,而且拥有良好的稳定性和循环使用性能。将其与GC-MS/MS相结合,建立了一种简单、高效、灵敏的检测7种PAHs的分析方法。该方法拥有检出限低、线性范围宽、重复性良好等优点。最后,将所建立的分析方法用于实际环境水样中PAHs的检测,成功地在实际湖泊水样中检测出了芴、菲、蒽、荧蒽和芘。综上所述,本实验中所建立的分析方法在环境水样中PAHs的分析和监测方面具有很大的应用潜力。
